# Feasibility and efficacy of simultaneous off-pump coronary artery bypass grafting and esophagectomy in elderly patients

**DOI:** 10.18632/oncotarget.14824

**Published:** 2017-01-26

**Authors:** Ban Liu, Chang Gu, Yuliang Wang, Xiaowei Wang, Wen Ge, Lingtong Shan, Yujian Wei, Xiaohan Xu, Yangyang Zhang

**Affiliations:** ^1^ Department of Cardiology, Shanghai Tenth People's Hospital, Tongji University School of Medicine, Shanghai, China; ^2^ Department of Thoracic Surgery, Shanghai Chest Hospital, Shanghai Jiao Tong University, Shanghai, China; ^3^ School of Public Health, Nanjing Medical University, Nanjing, China; ^4^ Department of Cardiovascular Surgery, The First Affiliated Hospital with Nanjing Medical University, Nanjing Medical University, Nanjing, China; ^5^ Department of Cardiovascular Surgery, Shuguang Hospital, Affiliated to Shanghai University of TCM, Shanghai, China; ^6^ The First Clinical Medical College of Nanjing Medical University, Nanjing, China; ^7^ Department of Cardiovascular Surgery, East Hospital, Tongji University School of Medicine, Shanghai, China; ^8^ Key Laboratory of Arrhythmias of the Ministry of Education of China, East Hospital, Tongji University School of Medicine, Shanghai, China

**Keywords:** esophagectomy, off-pump coronary artery bypass grafting, coronary artery disease, simultaneous, outcome

## Abstract

**Introduction:**

To analyze the outcomes of off-pump coronary artery bypass grafting (OPCABG) and esophagectomy simultaneously for patients with coronary artery disease (CAD) and coexisting esophageal cancer.

**Methods:**

Twenty-two patients with CAD and coexisting esophageal cancer underwent combined surgical interventions were subjected to the study. OPCABG was performed first, followed by esophagectomy. All the corresponding data including clinicopathological characteristics and postoperative outcomes were all investigated.

**Results:**

All the combined procedures were performed successfully. The average number of grafts was 2.36. Tumors were located at the middle third of the esophagus in 5 patients, at the lower third of the esophagus in 8 patients, at the esophageal gastric junction (EGJ) in 9 patients, respectively. The operations were carried out through a left lateral thoracotomy approach in 21 patients while a median sternotomy and left lateral thoracotomy approach was used in 1 patient for his condition rapidly worsened. Postoperatively, pneumonia occurred in 4 patients (18.2%). During the follow-up, three patients died of cancer metastasis /recurrence (6, 18, 37 months) and one died of pneumonia (1 month). The cumulative 5 years survival rate is 52.9%.

**Conclusions:**

The combined procedure of OPCABG and esophagectomy is a safe and effective treatment option for patients with severe CAD and esophageal cancer.

Esophageal cancer is one of the most common malignant disorder and cancer-related mortality, with an overall 1-year survival of 78% and a 5-year survival of 42% [1]. In China, it is estimated that the incidence and mortality rates of esophageal cancer were over twice as high as the rates in the world in 2012 [2]. Coronary artery disease (CAD) is the second leading cause of death in China, representing 13-22% of cardiovascular deaths for Chinese population [3]. The group of patients with tumors and coexisting CAD is common and is expected to increase due to an increasing aging population and improvement of diagnostic facilities. Although multi-modality treatment with surgery, chemotherapy and radiotherapy has been developed to improve the outcome for esophageal cancer patients, the esophagectomy is still considered to be the mainstay of potentially curative treatment [4]. It is very difficult to decide how to treat patients who have a resectable esophageal cancer and coexisting severe CAD. There are few studies reported the simultaneous operations of coronary artery bypass grafting (CABG) and esophagectomy, and most of them are case reports in selected patients [5–9]. Zhao et al reported a series study about simultaneous operation of severe CAD and esophageal cancer, only including six selected patients [10]. Fortunately, the remarkable improvement of surgical techniques makes it possible to conduct combined cardiac and malignant surgical operations. Simultaneous surgery of severe CAD and esophageal cancer in elderly patients was performed in our hospital and we followed up the effects of operations.

## RESULTS

### Clinicopathologic characteristics

The characteristics of the patients who underwent combined surgery are summarized in Table 1. There were 19 men and 3 women with a mean age of 65.64±6.67 years. The patients had mean 2.05 vessels disease. Tumors were located at the middle third of the esophagus in 5 patients, at the lower third of the esophagus in 8 patients, at the EGJ in 9 patients, respectively. The average number of grafts was 2.36.

**Table 1:** Clinical characteristic of the patients

Characteristics of patients	Range /Number	Means±SD/%
Age (years)	52-77	65.64±6.52
Sex (*n*)		
Male	19	86.36
Female	3	13.64
NYHA Class (*n*)		
II	22	100
Comorbidities (*n*)		
Hypertension	12	54.55
Diabetes mellitus	4	18.18
Smoking (*n*)	9	40.91
CAD Classification (*n*)		
Stable angina	18	81.82
Unstable angina	4	18.18
Number of disease vessels	1-3	2.05±0.77
Tumor location		
Middle esophagus	5	22.73
Low esophagus	8	36.36
EGJ	9	40.91
Preoperative risk evaluation		
SinoSCORE		0.74±0.35
STS		0.66±0.30
EuroSCORE II		1.76±0.76

**Abbreviations:** NYHA, New York Heart Association; CAD, coronary artery disease. EGJ, esophageal gastric junction; SinoSCORE, Sino-System for Coronary Operative Risk Evaluation; STS, the Society of Thoracic Surgeons; EuroSCORE II, the European System for Cardiac Operative Risk Evaluation II.

All the patients were underwent R0 resection. The average number of removed lymph node was 12.9 (2-39). Mean operation time was 404.73 min (250-585 min) while the mean intraoperative blood loss was 606.82 ml (200-1500 ml). Surgical outcomes are summarized in Table 2. Postoperative complications (pneumonia) occurred in 4 patients (18.2%). There were no deaths and no myocardial ischemia recurrent within 30 days after the surgery.

**Table 2:** Postoperative outcome of patients with lung cancer

Variables	Range /Number	Means±SD/%
Operation time (min)	250-585	404.73±72.51
Operation bleeding (ml)	200-1500	606.82±297.83
Grafts (n)	1-4	2.36±0.98
ICU stay (day)	0.52-2.76	1.31±0.63
Postoperative hospital stay (day)	11-37	19.59±6.04
Complications		
Pneumonia	4	18.2
Surgical approach		
Single incision approach	21	95.45
Two-incision approach	1	4.55
Location of anastomosis		
Below the aortic arch	14	63.64
Above the aortic arch	8	36.36

**Abbreviations:** LIMA: Left Internal Mammary Artery; SVG: Saphenous Vein Graft; ICU: Intensive Care Unit.

Pathological outcomes are summarized in Table 3. All patients had a histological diagnosis of esophageal cancer. Ten patients had squamous cell carcinoma, 8 had adenocarcinoma, 1 had endocrine cell carcinoma, 2 had adenosquamous carcinoma, and 1 had small cell carcinoma, respectively. After pathological examination, lymph node metastasis (+) was identified in 10 (45.5%) of the 22 patients.

**Table 3:** Pathological outcomes

Pathology of tumor	Range /Number	Means±SD/%
T size (cm)	0.6∼8	3.49±1.79
Pathologic type		
Adenocarcinoma	8	36.4
Squamous Cell Carcinoma	10	45.4
Others	4	18.2
Differentiation of tumor		
Low	3	13.64
Middle	15	68.18
High	4	18.18
Stage		
IA	2	9.09
IB	4	18.18
IIA	6	27.27
IIB	1	4.55
IIIA	6	27.27
IIIB	3	13.64
Lymph node metastasis		
Positive	10	45.45
Negative	12	54.55

### Follow-up

**Figure 1:**
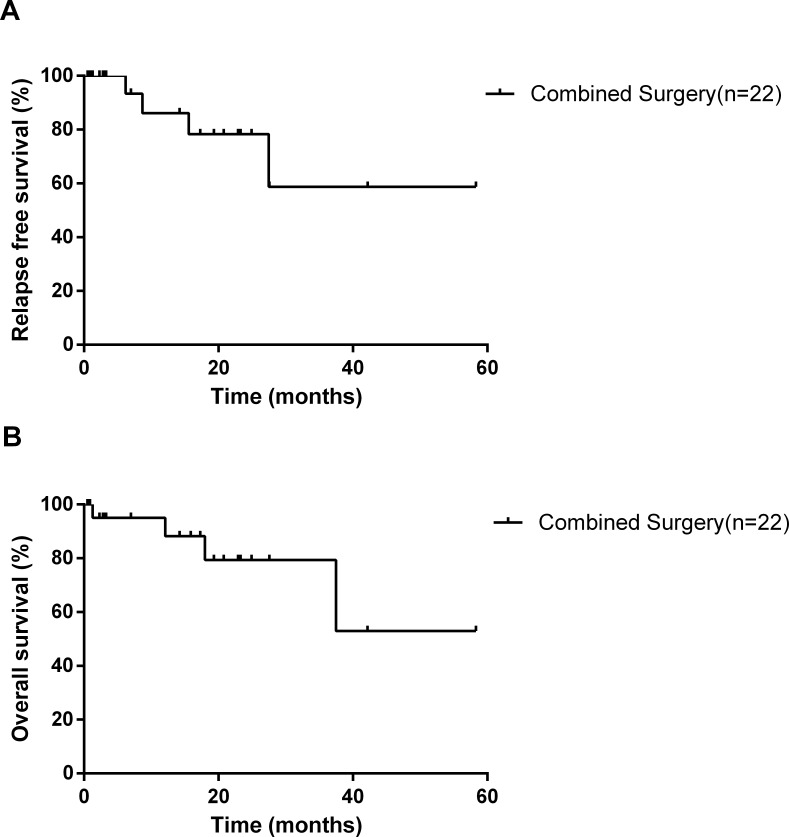
Kaplan-Meier survival curves for relapse-free survival **A**. and overall survival **B**. according to patients in our study who underwent simultaneous esophagectomy and coronary artery bypass grafting.

All the patients were followed up from 1 month to 58 months. In our series, seven patients were treated with chemotherapy only, one patient was treated with radiotherapy only and one patient was treated with chemoradiotherapy, respectively. Three patients died of tumor metastasis or recurrence (6, 18, 37 months after surgery, respectively), one died of pneumonia (1 month after surgery), but no patient died of cardiovascular events during the follow-up period. The remaining 18 patients survived from 1 month to 58 months during the follow-up. The median survival time is 17 months. The cumulative 5 years survival rate is 52.9% (Figure 1).

## DISCUSSION

In this study, we analyzed the outcomes of surgery for esophageal cancer in patients who had concomitant severe CAD that required operation. In China, the incidence of esophageal cancer are 14.73/100, 000 in 2012, which is about two fold higher than that in the world [11]. Esophageal cancer has a poor survival since patients are asymptomatic in early stages and usually diagnosed at the advanced stage [12]. CAD is the second leading cause of death in China. It is common that patients with esophageal cancer and coexisting CAD. CAD carries a significant impact on the surgical morbidity of patients with malignant disease [13].

Cardiac revascularization involving percutaneous coronary intervention (PCI) has a significant risk of major intraoperative cardiac ischemia 6-12 weeks after the stenting [14]. Patients with coronary stents also need intensive anticoagulation, which made them at high risk for hemorrhagic complications when undergoing thoracic surgery. Comparing the results of non-cardiac surgery after myocardial revascularization using either PCI or CABG operation, it was demonstrated that the incidence of myocardial infarction and mortality were higher among those patients who received PCI [15]. Patients with left main coronary artery disease or three-vessel disease also should generally be considered for CABG. In this study, the mean number of disease vessels is 2.05, and the average age of patients is 65.64years. It is reported that long-term all-cause mortality is similar between PCI and CABG managed patients, but PCI treatment related to higher rate of repeat revascularization [16]. When a coronary intervention is performed in a patient who is being considered for surgery soon after coronary intervention, efforts should be made to avoid stent placement if possible.

It is a very difficult issue to handle patients who need cardiac and malignant surgical operations at the same time, especially those resectable tumor patients with severe CAD, as contraindications to each other [17, 18]. Optimal treatment strategies for simultaneously diagnosed esophageal cancer and CAD remain debatable. The two associated procedures are performed simultaneously by a one-staged operation [19,20] or separately by two-staged operations [21, 22]. CAD leads to surgical morbidity of patients with malignant disease, and cardiac revascularization should be carried out first to avoid perioperative risk of myocardial ischemia or infarction and cardiac dysfunction [23]. In two-staged operations, cardiac revascularization is carried out first, and cancer resection is done secondly. If PCI is performed first, the intensive antiplatelet therapy usually needs for 3-6 months before cancer resection [24]. Adverse outcomes of cancer resection soon after PCI are associated with a major risk of operative myocardial ischemia [15]. At least 3 months delay between two operations has been recommended to minimize the risk of in-stent thrombosis [23]. However, the time lapse between cardiac and cancer surgeries is the major concern involving the two-staged operation, which may result in cancer progression [23].

One-staged operation managed both cardiac and non-cardiac diseases at the same session, and had no time delay in the treatment of the esophageal cancer. Both operations can be solved simultaneously avoiding a second operation. Simultaneous surgery has already been carried out in the 1980s for combined treatment of pulmonary neoplasia and cardiac surgical disorders. In one stage operation, CABG is performed first, considering cardiac surgery is an aseptic surgery, and cardiac revascularization is carried out first to avoid perioperative myocardial ischemia [25, 26]. Compared to a two-stage operation, a one-stage operation also has advantages including a single induction of general anesthesia, shorter overall hospital stay, and reduced cost of treatment [27]. Previous reports have demonstrated patients with lung cancer or esophageal cancer can be solved properly through a single incision [20, 28].

Conventional cardiopulmonary bypass and off-pump CABG techniques have been used successfully to revascularize the myocardium. The incidence of mortality and morbidity varied with each technique. Patients who underwent off-pump bypass had a 0% mortality, and 0-48% morbidity, in comparison to 0% to 20.8% and 0% to 67% among conventional bypass treated patients, respectively [26]. The off-pump bypass may avoid the detrimental effect of extracorporal circulation, which would affect tumor growth and dissemination [29, 30].

When patients’ conditions rapidly worsened, two separated incisions are needed to shorten the time of revascularization. Two separated incisions (median sternotomy and left lateral thoracotomy) were used in one patient in this study, who had persistent angina pectoris. With the surgeons’ experience, single incision (left lateral thoracotomy) was used to complete the combined surgeries. The cardiac surgery performed first, followed by the cancer resection, which is different from one recent study [10]. Considering our patients are elderly persons with severe CAD, cardiac surgery should performed first to decrease perioperative infection and cardiac incidence. In this study, all tumors located at the middle or lower esophagus. The left lateral thoracotomy incision was convenient to perform the off-pump CABG, well exposed the esophageal cancer and relative anatomies, and may have lower incidence of postoperative complications and shorter hospital stay [31]. In conclusion, the single left transthoracic approach was chosen for the following features: simple operation process, small trauma to the patient, and removal of the tumor or the lymph nodes to the greatest extent. The optimum extent of lymph node resection is significantly associated with long-term survival after surgery, transthoracic esophageal resection may effectively clean the number of lymph nodes [32, 33].

The internal mammary artery (IMA) is considered the best choice as the graft for CABG. The patients with simultaneous esophagectomy and CABG were also recommended IMA as the graft [9]. However, if IMA cannot be easily harvested as a graft, the saphenous vein could be an alternative [6]. The mixed application of arteries and veins is usually used in multi-vessel diseases. The CABG grafts are selected according to the coronary artery conditions, malignant degree of cancer and operative incision. In this study, the average number of anastomosed coronary vessels was 2.36, including 4 patients treated with IMA to the left anterior descending artery graft and 18 patients treated with saphenous vein grafts. The 10-year patency rate of saphenous vein graft is only 40%-60% [34]. However, it can ensure blood supply to the heart muscle and help to avoid occurrence of cardiovascular events within their life expectancy.

In our study, the hospital mortality was 4.5%. It is feasible and efficient to perform simultaneous surgeries on patients with both cardiac and non-cardiac diseases when considering the hospital mortality of 0%-6.5% as reported by other researchers [19, 35]. The wound complication was comparatively low. Postoperative complications (pneumonia) occurred in 4 patients (18.18%). All the complications were treated and all the patients were discharged from the hospital except the one died of pneumonia at 1 month after surgery. The simultaneous surgeries did not increase the surgical trauma and affect postoperative recovery [26]. The cumulative 5-year survival rate is 52.9% in our series. Five year survival rate was 42% in single esophagectomy patients with negative lymph node [1]. In this study, about half patients (45.45%) had lymph node-positive esophageal cancer, and received combined surgeries.

This study has some limitations. This is a retrospective and not randomized controlled design, and relative small number of patients is included in this study. Furthermore, a potential problem existed in the study was postoperative thoracic cavity infection, because esophagectomy was type II incision operation while OPCABG was type I incision operation, the two types of surgery were operated with the same incision, which would cause severe postoperative thoracic cavity infection even our surgeons followed the principles of antisepsis. Based on this, to prevent postoperative infection, first, use prophylactic antibiotics, then, OPCABG should be operated preferentially, followed by esophagectomy, and finally, improve the management of respiratory tract, wound and drainage tube.

In conclusion, the simultaneous operation is safe and technically feasible, based on observed surgical outcomes and complication data.

## MATERIALS AND METHODS

Twenty-two patients with the diagnoses of both esophageal cancer and CAD were operated on between September 2010 and August 2016. Myocardial revascularization was performed simultaneously with the esophagectomy. Patients’ data included age, sex, smoking history, disease stage, and comorbidities, including history of hypertension, diabetic mellitus, and chronic obstructive pulmonary disease. Cancer staging was based on the American Joint Committee on Cancer (AJCC) staging manual (7th Edition) [36]. In our study, tumors 25-30 cm and 30-40 cm distal to the incisor were considered as middle third and lower third thoracic locations, respectively. Tumors were located at the esophageal gastric junction (EGJ) in 9 patients, at the lower esophagus in 8 patients and at the middle esophagus in 5 patients, respectively. All patients received coronary angiography and fiber gastroscopy with histology of the biopsy specimens to confirm the combined heart and esophageal disease. Postoperative variables included major morbidity rate, ICU stay, hospitalization time and in-hospital death. All patients received routine clinical examination, blood serum analysis, chest CT, electrocardiogram and abdominal ultrasound.

### Surgical procedure

The patient was placed in the right lateral decubitus position. A traditional posterolateral incision was made along the sixth or seventh intercostal space in the left hemi-thorax for resection. In the first stage of the operation, cardiac revascularization was performed on the beating heart (off-pump CABG), followed by the esophageal cancer resection. If the mammary graft was difficult to dissect through the left lateral incision, the saphenous veins were used as the bypass grafts. Systematic mediastinal lymphadenectomy was performed with anatomical dissection of the esophagus. The gastric conduit was then harvested via transdiaphragmatic approach along with abdominal lymph node clearance. Construction of the anastomosis was done by stapling anastomosis. Patients with EGJ cancer and/or lower third esophageal cancer had the anastomosis done below the inner aspect of the aortic arch. Patients with middle third esophageal cancer had esophagogastrostomy reconstruction done above the inner aspect of the aortic arch. A single left thoracotomy incision approach was employed for 21 of the patients. The off-pump CABG was performed and the feeding vessels were anastomosed to the descending aorta. Besides, one patient was operated by two separated incisions (median sternotomy and left lateral thoracotomy), whose condition rapidly worsened, off-pump CABG was performed first and the saphenous veins were anastomosed to the ascending aorta. Regarding to anticoagulant therapy, low molecular weight heparin was given until their discharge from hospital depending on the blood loss and body weight. Plavix or Brilinta was commenced after stopping low molecular weight heparin.

### Follow up

All patients were followed up in the clinic visits at the following intervals: the first after 1 month, the second at 3 months and then at 6 monthly intervals. All patients received the following tests at each follow-up visit: standard clinical examination, electrocardiogram, cardiac echo, and chest X-rays. After the initial 6 months, and then followed up every 6 months until the third year after the operation.

### Statistical analyses

Statistical analyses were carried out using SPSS software version 16.0 (SPSS Inc., Chicago, IL, USA). Continuous variables are presented as mean ± standard deviation. Kaplan-Meier method was used to make a statistical description of the survival time.

## Abbreviations

OPCABG: off-pump coronary artery bypass grafting
CAD: coronary artery disease
PCI: percutaneous coronary intervention
AJCC: American Joint Committee on Cancer
EGJ: esophageal gastric junction
IMA: internal mammary artery
